# Editorial

**Published:** 1864-05

**Authors:** 


### Editorial.

---

ILLINOIS STATE MEDICAL SOCIETY.—Before this number of the EXAMINER will reach its distant readers, the Annual Meeting of this Society will be in Session in this city. We are hoping for, and anticipating a full meeting. The Committee of Arrangements have made their programme for a session of three days at least. The Illinois State Medical Society has gained the reputation abroad, of being one of the most active State organizations in the country. We are confident this reputation will be increased by the results of the present meeting. We shall give a full record of its proceedings in the next number of the EXAMINER.

THE SPRING AND SUMMER TERM IN THE CHICAGO MEDICAL COLLEGE.—The summer course of instruction in this institution is progressing with regularity and interest. There is a class of excellent young men in attendance, and the advantages they enjoy could hardly be excelled. They have four regular clinics in the Wards of the Mercy Hospital each week, under the charge of Professors DAVIS and ANDREWS; two in the College Dispensary, under the care of Professors ANDREWS and BYFORD; and one in the Soldiers' Home, under care of Professor HOLLISTER. In addition to these ample facilities for gaining practical knowledge, they have from one to two Lectures and Examinations in the various branches of medical science daily, with dissections, and access to the reading-room and museum of the College.

---

MORTALITY AMONG THE INTERNES OR RESIDENT PHYSICIANS IN HOSPITALS.—We learn from our exchanges that during the past year, and indeed during several of the past years, there has been a large ratio of mortality among those engaged as Internes, Assistants, or resident physicians in the Bellevue Hospital, New York, as well as in some other large general Hospitals. We observe that this high degree of mortality has resulted chiefly from continued fever; and has been attributed, by some, to the admission of many cases of Typhoid and Typhus fevers into the same wards with the sick of other diseases, and the consequent diffusion of the fever poison throughout all departments of such institutions. Without disturbing, at present, the question concerning the specific contagiousness of continued fevers, we are confident that nearly all the mortality among the students and Assistants in Hospitals and Colleges could be prevented by proper attention to matters of personal hygiene. During the last fourteen years we have been on duty constantly as Attending Physician to a General Hospital, into the wards of which, both Typhoid and Typhus fevers are freely admitted. During all the time, either advanced students or recent graduates have been employed as Internes or Assistants, boarding and lodging in the institution; and large classes have been daily admitted into the wards for clinical instruction. Of all those employed as Internes or resident Assistants, not more than four have suffered attacks of continued fever, and none of them have died. From personal observation, we are satisfied that all those who suffered attacks, neglected free exercise in the open air, and their sleeping-room was in the basement or lower floor of the Hospital. Again, during all the fourteen years past, classes of students varying from 20 to 75 in number, have visited the hospital for clinical instruction, at least four times a week, during each annual period of Medical College instruction. They have surrounded the beds of continued fever patients in every stage of the disease as freely as those of any other class; but in no instance have we been able to trace an attack of fever among them to such intercourse. During the first three years of our connexion with a medical college, we found each year, when about one-third of the session had passed, a considerable number of the students began to complain of the premonitory symptoms of typhoid fever. In only a small proportion of the cases did the disease assume any severe form, and in only one case did it terminate fatally. Its frequent occurrence, however, caused us to inquire carefully concerning the circumstances under which it was developed. We found that in almost every instance the students attacked were lodging in small, ill-ventilated rooms, taking no out-door exercise except simply from their rooms to the college and back, and often reading late at night. The connexion between these circumstances and the attacks of sickness, was so obvious that we have ever since embraced the opportunity, at an early period in each College term, to call the attention of the classes to them, and to insist that every student should take a reasonable amount of active, vigorous exercise in the open air, and keep his lodging room well ventilated. Since we have adopted this course, eleven years of College and Hospital instruction have passed by, and not a single member of the classes attending the institutions with which we are and were connected, has died from continued Fever, whether Typhoid or Typhus. So important do we deem open air exercise for those who are daily crowding the wards of Hospitals and the Lecture-rooms of Colleges, for instruction, that in selecting a location for the Chicago Medical College building in this city, we purposely placed it at such a distance from the Mercy Hospital as to require a brisk walk of from *ten* to *fifteen* minutes to pass from the clinical wards of the latter to the Lecture-rooms of the former. This walk, often converted by the students into a playful run, most effectually dispels whatever of deleterious miasm or infection they may have imbibed in their contact with the sick. We are fully satisfied that, while every Medical College should embrace an Hospital as a necessary part of its system of medical instruction, a due regard for the health of the classes who attend, forbids that both should be under the same roof. If the medical officers of Hospitals and the faculties of Colleges would more rigidly require active out-door exercise and thorough ventilation of lodging rooms, on the part of internes and students, we are quite sure much sickness and many deaths would be prevented.

---

The New York Medical Independent and Pharmaceutical Reporter.—This is the title of a new medical Journal published in New York. It is issued every week, and each number contains sixteen pages: Price, two dollars per annum. Publishing-office, 447 Broome Street.

We welcome the *Independent* to our exchange list, and should doubtless welcome the Editors into the field of medical journalism, if we knew who they were.

---

Preservation of Gum and Starch Paste.—By John M. Maisch. The paste made by gum tragacanth and gum arabic, which is so extensively used by the apothecaries in this country, acquires, particularly during the warm season, a very unpleasant and even offensive odor in consequence of fermentation, which soon commences on exposure to the air. Oil of cloves, alum, and other essential oils and salts, are frequently added to counteract this tendency, with but partial success, the volatile oils merely hiding to a certain degree the offensive odor developed, and retarding the fermentation incompletely. For some time past I have availed myself of the antiseptic property of creosote, which may be added to these pastes recently made, until its odor is faintly apparent. The result is their perfect preservation, no offensive odor being disengaged, and their adhesiveness is not impaired by keeping them for months.—*Am. Journ. of Pharm.*

---

TERCHLORIDE OF CARBON.—Mr. Bryant informs us that at Guy's Hospital the terchloride of carbon has been employed for many years, and that it was a very favorite remedy of the late Mr. Ashton Key, tracing its employment back, therefore, at least fifteen years. As a lotion it has acquired considerable value, and may be looked upon as a stimulant, and in a measure as a disinfectant. In sloughing and fetid ulcers it is of great use. It may be used in the indolent and weak ulcer with general advantage. The usual strength of the lotion is from *m*xx to ʒss of the drug to an ounce of water. It has an agreeable odor and rapid effect. In cases of gangrene, and in sloughing phagedena, it may be employed in its concentrated form with some confidence, a wound thus affected rapidly taking on a more healthy aspect. Upon the whole, it is a very valuable local stimulant, and in Mr. Bryant's estimation ranks above most of the drugs of that class now in use.—*Med. Times and Gaz.* and *Med. News.*

---

GALVANISM IN TETANUS.—The celebrated Italian physiologist, Matteucci, has addressed a communication to the French Academy of Sciences upon the employment of the continuous electrical current in the treatment of tetanus; and in a note to M. Flourens he earnestly begs that the attention of physiologists and physicians may be turned to the subject, as he firmly believes that a therapeutical procedure will result, which, if it do not effect a cure in this terrible disease, will, at all events, produce great diminution of suffering. Seeing recently, in an American journal, the fact stated, that the continuous current had been advantageously employed in a case of hydrophobia, he called to mind a case of tetanus published many years since by Nobili and himself. It is well known that a condition of tetanic contraction may be excited under two circumstances, viz., the interrupted passage of the electric current into the nerves and muscles of an animal at very short intervals, and the continuous passage of the current into the nerve in the opposite direction to its ramifications. It has been the object of some of Professor Matteucci's communications in the *Philosophical Transactions*, to explain how this is brought about by the production of secondary polarities. What, however, we have to do with at present is the fact that a nerve which has in this way acquired the property of exciting tetanic contractions, instantly loses such property as soon as it is submitted anew to a continuous current. Reasoning from analogy, it was thought that tetanus might be assimilated, as regards the state of the nerves, with the condition of an animal in which interrupted currents or a continuous inverse current have been employed; and the hope was entertained that a direct continuous current would produce the cessation or diminution of the contractions in the one case as in the other. And so in effect it was found that the patient, while he was subjected to a continuous electric current from 30 to 40 pairs of plates, no longer suffered the same violent convulsions, and was able to open and shut his mouth. This amelioration continued during several minutes, after when the contractions returned, notwithstanding the passage of the current was suspended awhile, and then reproduced with from 50 to 60 pairs. Amelioration again followed; and these alternations continued during several hours, the salutary effects of the current gradually diminishing, and at last ceasing entirely.—*Med. Times and Gaz.*

---

ANÆSTHESIA FROM CHLOROFORM PROLONGED BY THE HYPODERMIC INJECTION OF MORPHIA.—Translated by Dr. HOMBURG, Cincinnati. The following observations of Professor Nupbaum, of Munich, are likely to prove of vast importance not only in chirurgical, but also for internal medical treatment, for instance, in reference to the therapy of the tetanus, various neuroses, etc., yea, even in experimental physiology. Since it appears to us desirable that the valuable experiments in question, should be confirmed by other surgeons and physicians so that experiments may be had in the most varied manner, we hasten to communicate them briefly, even without waiting for a greater number of cases bearing thereon.

Professor Nupbaum removed, about three weeks ago, from a patient aged forty, a miller, residing in Foelz, a great sarcomatous tumor on the neck, using chloroform in the usual manner. To silence pains after the operation, which required a complete separation of plexus cervicalis, he injected beneath his skin, while still under the influence of chloroform, one grain of acetate of morphine. The person operated upon did not subsequently—as usual—awaken from his narcotism, but slept on, breathing regularly and calmly, uninterruptedly, for twelve hours. He endured during this sleep the deepest stitches of the needle, incisions into the skin, and the application of red-hot iron, etc., without even the slightest reaction against the same. Finally, he awoke from deep slumber, exactly as if he had just passed through a chloroform narcotism.

A few days later, Prof. Nupbaum most pleasingly surprised at this exhibition, and the effect just stated of subcutaneous application of morphine on a second patient, a Mr. M., in Swabia, upon whom, in consequence of a cancer, he had just executed the resection of the upper maxillary bone without removing the alveolar process during the chloroform narcotism, and had finally, on account of cancerous irritation in the facial skin, undertaken a transplantation in the neighborhood of the temples and forehead by closing the wound. This patient, too, slept with complete absence of all feeling during eight hours amid the most quiet breathing. His pulse remained in rhythm and number perfectly regular. The effect of the narcotic appears the more surprising in this case, because the same dose of acetate of morphine had a few days previous been injected hypodermically without producing sleep, and still less anæsthesia.

Two other cases embrace a woman fifty years old, and a seven year old boy, upon both of whom only about half a grain of morphine had been subcutaneously injected; and both slept from five to six hours the same quiet sleep, and enjoyed an equal anæsthetic condition. Another case, in which the experiment in question failed, has up to now not been observed by Professor Nupbaum.

From the preceding observations appears to arise a physiological experimental point, that must on further use tend doubtlessly to most gratifying results. Obviously it appears as if the hypodermic application of morphine, and perhaps of other narcotics, for instance, of atrophia, might during the chloroform narcose preserve for several (six to twelve) hours that peculiar condition of the central nervous system, of which we know—it is to be lamented—as yet so little, and which is temporarily produced by the effect of inhaled chloroform, and to do this by greater or lesser doses of morphine; as long at least as the effect of morphine is maintained; and of course also the arracothesy, which to produce through the inhalation of chloroform is, as well known, one of the most beneficent inventions in aid of suffering humanity.—*Cincinnati Lancet & Observer.*

---

Treatment of Tendinous Rheumatism by the External Employment of Sulphur.—Tendinous rheumatism, according to Dr. Renard, differs from acute rheumatism by the absence of the general symptoms, and from the chronic by the presence of local inflammatory symptoms. Dr. Renard suffered from this complaint himself after an attack of acute rheumatism, for which he was copiously bled. The parts affected were the tendons of the hamstring muscles, and no improvement resulted after a long course of diaphoretics, camphor, terebinthinate, and other liniments, and the administration of the solanaceæ. At last Dr. Renard saw a passage in an English medical journal, stating that persons suffering from rheumatism in the legs had only to dust the inside of their stockings with sulphur. He immediately employed this simple remedy, the sulphur being the commercial flowers of brimstone, which contain some sulphurous acid. The curative effect was very well marked, for Dr. Renard walked in the evening, then renewed the sulphur in the stockings before sleeping in them, found himself very much relieved the next morning, and nearly quite cured on the morning after. A few days later, he left off the brimstone, and the pain reappeared in the soles of the feet, but yielded very soon to the reapplication of sulphur. Since the year 1857, when he was first attacked, the same experiment was repeated every winter when he was suffering from chronic tenodynia, either in the hams, the heels, or the elbows. He felt under the influence of the contact of the flowers of brimstone, the skin becoming hotter, slightly excited, and more disposed to sweating; and, as soon as this effect was produced, the relief of the pain seemed to be immediately marked. Whatever may be the explanation of the manner in which sulphur exerts its curative agency, Dr. Renard affirms that it has a beneficial effect upon the rheumatic pains of the tendons, and that this action is the more rapid and certain in proportion as the tendons are more superficial and the sulphur is kept more closely over the painful parts.—*B. & F. Med. Chir. Rev.*, Jan. 1864, from *L'Union Medicale*, April 21, 1863.

---

Traumatic Tetanus successfully treated with Chloroform and subsequent Use of Belladonna.—Dr. L. C. Lord reports (*San Francisco Medical Press*, Oct. 1863) the following case: Some weeks since, in the St. Mary's Hospital in this city, there was admitted a young man with fracture of the os femoris in its upper third; the fracture, which was comminuted in character, was the result of a fall from one of the city cars, while in motion.

The injury was treated by the application of Desault's longextending and counter-extending splint. Shortly after the limb was dressed in this manner, tetanic symptoms presented themselves in the form of trismus, which ultimately became general, the whole body being thrown into violent muscular contractions. Soon after the supervention of these symptoms, the patient was put under the influence of chloroform by inhalation. He was maintained in a state of constant anæsthesia for near seven hours, consuming, in the meantime, several ounces of chloroform, administered by means of an inhaler, so constructed, that but a small amount of the article could escape without being breathed. After the use of chloroform for that length of time, the tetanic symptoms so far disappeared, that the inhalation was suspended, and the patient was ordered belladonna; opiates were also given him. On the following day, trismus again ensued, when resort was had again to the chloroform. The closure of the lower jaws was quickly relieved, whereupon the inhalation was discontinued.

The remedy to which I am inclined to refer the rescue of the man's life in this case, was chloroform. The inhalation, as will be perceived, was carried to a much greater extent than usual, or than prudence would dictate in any other than a hopeless case. After the discontinuance of the anæsthetic, the patient presented symptoms of aberration of mind, which were present for several days afterwards, though they gradually became less, and, in a week afterwards, they disappeared. The patient is yet under my charge, in every respect doing well, though time enough has not yet elapsed to have effected entire union of his fractured femur.

---

Hospital Gangrene.—Dr. Frank H. Hamilton, Jr., Asst. Surg. U. S. A., has given (*American Medical Times*, Oct. 31, 1863) a tabular statement of 33 cases of hospital gangrene which occurred in the McDougal General Hospital. It appears from this table that but two of the cases terminated fatally, and these some days after the gangrene had been arrested. In one of these the patient died from exhaustion, the result of extensive suppuration in the knee-joint, the wound having been in a perfectly healthy condition for several days. In the other the patient died from dysentery, his wound having put on a healthy action two weeks before his disease.

In one case where nitric acid was used, the disease was not arrested, and at the end of ten days it was found necessary to amputate the leg above the knee. The stump healed by the first intention. An analysis of the table shows that the average duration of all cases, under all treatments, amounts to 12.15 days.

| | |
|---|---|
| Number treated with nitric acid . . | 18 |
| Average duration of disease . . . | 16 days. |
| Number treated with sol. bromine . . | 14 |
| Average duration . . . . . . | 6.6428 days. |
| Number treated with iodine . . . | 1 |
| Average duration . . . . . . | 7 days. |

These results are decidedly favorable to bromine.

---

VARICOSE VEINS.—Mr. SKEY remarked (*Lancet*, Jan. 2, 1864) that the treatment of varicose veins involves two objects: "1st, the increase of power to these organs; and 2d, the turning the current of the venous circulation into healthier channels. The first is effected by the liberal administration of nutrio-stimulants. The second object has tested the inventive faculties of many surgeons. I leave it to others to commend the various schemes adopted by them. I discountenance, from long observation of its incompetency to cure, the employment of the needle, whether through the vein or under it, single or double. It has these objections: 1st, it is not unattended with danger; and 2d, it fails to obliterate the vein, except at the point of its application, mainly because the applications cannot be safely made in numbers proportionate to that of the veins affected. I have at present in St. Bartholomew's Hospital a woman under treatment for varicose veins of the leg, whose limb was jeopardized by the employment of the needle a year ago. A long illness, with severe inflammation and extensive abscesses, followed. The same limb is again under treatment for the original disease. There is no danger in making any number of small eschars on the most projecting surfaces of varicose veins, if made with an escharotic composed of two-fifths of pure potash and three-fifths of powdered lime. This powder, well combined, is made into a paste with alcohol. Whether other escharotics are dangerous in their operation on veins, I do not stop to inquire; I only know that the Vienna paste, combined as I have above described it, is not. These eschars may be made in any number proportionate to the extent of the disease. I have treated perhaps 250 cases in the course of the last ten years, and I continue to treat them, by the same means. The paste is applied over the most projecting parts of the vein in the following manner: through a series of about four layers of adhesive plaster a circle is cut of the size of a threepenny-piece or smaller. The influence of the escharotic extends through the vein; and it is curious to observe that from the hour of its application the entire vein appears to be obliterated, and is undetectable to the finger on pressure. From ten to twenty-five eschars may be applied between the ankle and the knee. Twenty minutes suffice for the full operation of the escharotic, and an average of one month for the cure. In very weak constitutions the ulcers will heal very slowly, unless well-directed efforts be made to give force to the general system."

---

Structure of Indurated Chancre of the Prepuce.—In a paper read before the Biological Society of Paris, M. Ordonez has given the following summary of the appearances observed by him on making a histological examination of indurated chancre. 1. The epidermis is considerably thickened around the ulcerated part. The most superficial cells all present a central nucleus, tolerably large, with from one to four nucleoli; contrary to what is met with in healthy epidermis, where the cells lose their nuclei as they approach the external surface of the skin. 2. The interpapillary digitations in the true skin are larger at the level of the chancre than in the healthy skin. The epithelial cells are very closely packed, larger than in the normal state, and infiltrated by a very transparent fluid, coagulable by alcohol. 3. At the level of the papillary layer of the skin, small hemorrhagic clots may readily be detected, produced, no doubt, by the rupture of the small capillary loops distributed in the papillæ. Hæmatosine, mixed with red corpuscles in various stages of change, is effused in patches, between the papillary and the mucous layers. 4. The meshes of the cutis vera, from the papillary layer to its deepest portion, are infiltrated with a large quantity of plastic lymph. On merely making thin slices of the chancre, a large quantity of a very transparent, slightly viscid fluid, coagulating slowly on contact with the air, may be made to escape by pressure or by the action of the cutting instrument. This liquid, examined microscopically and with the aid of reagents, appears to be plastic lymph, or blastema. 5. The papillæ are increased in size, without being altered in shape. They are infiltrated with a large number of embryonic or transitory elements of the fibrous or connective tissue. These consist of round or oval nuclei, varying in diameter from .00016 to .00028 and .00036 of an inch; of small fusiform, fibro-plastic bodies in an ordinary state of evolution; and of small bundles of fibres of fibrous or connective tissue in progress of formation, and still presenting nuclei. 6. In the substance of the derma are to be found a number of fibrous cords, with perfectly developed fibres, and presenting a brilliant white aspect, contrasting remarkably with the adjacent tissue. This appearance is best presented by recent sections of the induration, examined by the aid of distilled water; it is also present, but less distinct, in specimens that have been preserved in alcohol or glycerine. M. Ordonez thinks that the alterations in the skin which he has described, satisfactorily explain the peculiar induration characteristic of the infecting chancre.—*Gazette Medicale de Paris*, 11 Octobre, 1863.

---

SUBPUBIC PUNCTURE OF THE BLADDER.—To avoid the danger of peritonitis, which sometimes follows the operation of puncturing the bladder above the pubes, M. Voillemier has devised the following operation. The patient is placed on his back, with the legs slightly separated; the pelvis is raised by a thick cushion, so as to bring the pubes forward, and to prevent the distended bladder from embarrassing the operator. An assistant, standing at the left side of the patient, draws the penis downwards and backwards. Sitting at the patient's right side, the surgeon feels with his right fore-finger for the suspensory ligament, and with his left hand he introduces by the side of this ligament a trocar, curved so as to pass round the pubic bone. During this stage of the operation, the instrument is carefully supported and guided by the right hand, lest the trocar should turn too suddenly and come into contact with the bone. The canula having entered the bladder, the trocar is withdrawn. The operation was successfully performed by M. Voillemier, in the Hospital St. Louis, on October 14th. The cicatrization of the wound was complete in forty-eight hours; and, at the time of reporting, no trace of the operation remained, beyond a fibrous cord indicating the passage of the instrument.—*Brit. Med. Journ.*, Jan. 23, 1864, from *Gaz. Med. de Paris*, 14 Nov., 1863.

---

DIET IN DIABETES.—Dr. EDWARD SMITH concludes some interesting observations on this subject with the following summary of the proper diet in diabetes:—

1. *Fluids.*—To be limited by degrees daily until they shall not exceed five pounds and a-half in both fluid and solid food. Of this quantity two or three pints should consist of new or skimmed milk, and one pint, or less, of tea. In the cold season and at night they should always be given when hot. Of all alcohols brandy is the best, and may be given with water only, or added to milk, or beat up with egg and milk, and given several times daily. No fluid should be given in greater quantity than half a pint at a time; and, when milk is reduced in volume by cooking, the daily quantity of fluid must be made up by an additional supply of the same or other fluid.

2. *Solids.*—Dr. Prout's combination of eggs and milk (with sharps substituted for bran) is excellent. Four ounces of sharps and 4 oz. of peas, beans, or lentils may be made into bread or pudding, with milk, or into omelettes with eggs and herbs. Eggs and gelatin may be given when starchy food cannot be altogether intermitted. Eggs, gelatin, cheese, gluten, bread, meat, fat, and oils may be given as largely as they can be digested. The free use of salad oil should be urged, whether in the cooking of fish or flesh, or in the use of water-cress as a salad or drunk alone, so that several ounces may, if possible, be consumed daily; but as there are in all persons preferences and dislikes in reference to particular fats, that kind—whether butter, suet, oil, or fat of meat—should be allowed, which is the most agreeable. Four oz. of sharps, 3 oz. of wheaten flour, 5 oz. of peas, 1 lb. of meat, 2 oz. of cheese, 2 pints of milk, and 3 eggs, will afford more than about 13 oz. of carbon and 1 oz. of nitrogen daily.—*Lancet*, Feb. 6, 1864.

---

PRACTICABILITY OF ARRESTING THE DEVELOPMENT OF EPIDEMIC DISEASES BY THE USE OF ANTI-ZYMOTIC AGENTS.—Dr. ROBINSON read a paper on this subject before one of the sections of the British Association for the Advancement of Science, at its meeting at New Castle.

The author commenced by referring to the circumstance of the analogy between many of the phenomena of fevers and other zymotic diseases, and the ordinary process of fermentation having been perceived and recognized by Hippocrates and the oldest writers on medicine. Their idea was that a poisonous ferment, existing in the atmosphere, entered the mass of blood and induced in it a series of changes, which gave rise to the excessive heat, and other peculiarities of that class of diseases—at the present time, this doctrine, modified by the discoveries of Liebig and other chemists, has been adopted by most physicians, and forms the basis of the classification of disease framed by Dr. Farr, and used by the Registrar-General. It thus supposes living germs to exist in the atmosphere, which, when introduced into the body, give rise to a specific and regular series of morbid actions, pursuing a definite course in a definite time, as in small-pox—those germs being disclosed and multiplied, and producing others capable of reproducing in other bodies the same succession of changes—other lethologists have supposed that the atmospheric poison acts on the blood chemically, by giving rise to what may be termed catalytic actions—while the author is disposed to believe, from what he saw during the cholera epidemic in Newcastle in 1853, that some of these volatile organic matters in the atmosphere are capable of acting on the human body as direct poisons, and that this inanimate volatile poisonous matter also furnishes nutrition to the organic germs suspended in the air. After these preliminary remarks, he proceeded to refer briefly to a number of scattered facts, which seemed to him to indicate the existence of a great principle, which might hereafter be found applicable to the prevention or mitigation of epidemic diseases by the direct use of substances capable of arresting the process of morbific fermentation. He mentioned the following facts as converging to this conclusion: 1. Antiseptic substances, ranging from simple innocuous matters, such as sugar, up to the powerful metallic poisons, such as corrosive sublimate, and forming a very numerous and diversified group, have long been known to be capable of arresting the putrefaction of animal and vegetable structures. 2. The same substances prevent the formation of fungi, as is seen in the use of solutions of metallic salts in the taxidermy in the prevention of dry rot, &c. 3. Many of those agents are known to arrest at once the process of fermentation, as, for instance, sulphurous acid, and Emi and other chemists have observed under the microscope the rapid stoppage of the vitality of the yeast plant when a solution of arsenious acid was added to the fermenting liquor. 4. The formation of the fungus in and on the plant, which causes the vine disease, is prevented by applying sulphur to the affected vines. 5. In Cornwall it is believed that the arsenical fumes from the tin-calcining furnaces exercised an influence over the potato-plants in the neighborhood, which preserved them from the disease then affecting other parts of the same county. [A statement to this effect, signed by Captain Charles Thomas, Sen., of Dolwath, and sixteen cottagers, was here read.] 6. It has been found that when a species of fermentation has taken place in the human stomach, resulting in the development in large quantities of a minute organism (the sarcina ventriculi), this morbid action can be controlled and stopped by the direct anti-zymotic influence of certain salts, such as sulphate of soda, in doses perfectly compatible with the patient's safety. 7. In different parts of the world, among different races, a belief has long existed that certain antiseptic substances, of which arsenic may be taken as the type, are capable of acting as antidotes or preservative and curative agencies against atmospheric and other poisons, and in some cases that popular belief has proved to be well-founded. The experience of the multitude discovered the value of arsenic as a cure for ague long before it was recognized as such by physicians. The arsenical fumes of certain works in Cornwall were stated by the late Dr. Paris to have stopped the ague, previously endemic there. More recently it has been stated that the arsenic eaters of Styria are peculiarly exempt from fevers and other epidemic diseases. And in India the natives have long used arsenic as an antidote for the poison of snakes. Dr. Robinson concluded by expressing a belief that these scattered observations were not only sufficient to justify and necessitate further inquiries in this direction, but seemed in themselves to shadow forth the outline of a great law which might at some future time be productive of immense benefit to mankind.—*Med. Times and Gazette*, Sept. 26, 1863.

---

The Origin of Cow Pox and the Nature of Vaccine Virus.—Investigation on this subject in the Paris Academy of Medicine, has led to the following conclusions:—

1st. That vaccine virus (as a thing separate and apart) has no existence.

2d. That the pretended vaccine virus, which we consider as antagonistic to, and neutralizing the variolus virus itself.

3d. That the èquine and bovine species are subject to an eruptive malady, which is identical as regards its nature, with variola of the human species.

4th. It is demonstrated that the same is the fact as regards several other species of animals, pigs, sheep, dogs, goats, apes, etc.

5th. The local and general phenomena with animals is the same as those observed in man. The only difference as regards the pustules are those which depend on the structure of the skin and the number of the hairs.

6th. As in the human species, so in the equine and bovine, variola may appear sporadically or epidemically.

7th. From the horse we may inoculate the cow, and reciprocally.

8th. From the cow we may inoculate, without difficulty, individuals of the human species, provided they have not had spontaneous or inoculated variola.

9th. The cow, the horse, and several other species may be inoculated with variolus matter from the human species.

10th. When a variolus epidemic occurs among men, it often extends itself, by contagion, to other animals.

11th. An epidemic of variola may commence among animals, and extend to man.

12th. Inoculated variola produces a much less degree of general reaction, than does variola developed by contagion. This is true in both man and lower animals.

13th. The pustules which result from inoculated variola, are often limited to the points inoculated.

14th. When a secondary eruption is produced, it is almost always insignificant, and composed of a small number of pustules.

15th. In a general manner we may say that the variola of animals is more discrete, and less severe, than that of the human species.

16th. That the dangers of inoculation of variola in man have been much exaggerated. The unprejudiced study of what has been written on this subject will convince of this.

17th. It is probable that animals, as man, are subject to aphthous eruptions.

18th. But the *maladie aphtheuse*, as it is described by writers on veterinary medicine, is nothing else than variola.—*Medico Chirurgical Review.*

---

Divided Tendo-Achillis united by Silver Wire.—Dr. G. L. Simmons, of Sacramento, relates (*Pacific Med. and Surg. Journ.*, Jan. 1864) a case in which the tendo-Achillis of a man was completely severed accidentally about an inch from its attachment. Dr. S. found the upper edge of the cut tendon retracted an inch and a quarter into its sheath. Dr. S. flexed the limb, drew down the retracted tendon by strong forceps, and united the cut ends with a large-sized silver ligature; the leg was kept flexed for a few days with adhesive straps, after which the usual slipper and dog-collar were used. In a few weeks the patient was able to walk in a high-heeled shoe with but little pain. Scarcely any stiffness resulted from the injury, and at the date of the report he could walk freely with the slightest perceptible halt. The "propriety of using silver wire in uniting tendons," Dr. S. says, "can hardly be questioned. In the above case the result was all that could be desired; and, although position alone might possibly have accomplished the same end in the same time, yet it is probable that the perfect approximation of the parts by the ligature assisted in defining the bond of union until it became strong. In this case, after the uniting mass was perfected, I removed the silver wire, as the play of the tendon caused the foreign body to slightly irritate the neighboring tissues.

---

Treatment of Whooping Cough by Belladonna and Sulphate of Zinc.—E. Garraway, writing of whooping cough, says:—The preponderance of opinion is in favor of its being a nervous disorder; and appears to have as much claim to be so considered as asthma, chorea, epilepsy, or other convulsive disorders which it has been impossible to localize.

The treatment by belladonna and sulphate of zinc, in some fifty or sixty cases, was entirely successful: it was given in extract, either diffused in water with the zinc, with sufficient syrup to make it agreeable to children, or, to those who were old enough, in pills;—the dose being from one-sixth to one-fourth of a grain, of extract of belladonna, and half a grain of zinc, three or four times a day, steadily increasing the amount till, at the end of three weeks, children would be taking from four to six grains of belladonna, and twice that quantity of sulphate of zinc, daily.

So far as investigations went, it would appear that both the tolerance of the remedy and the speedy subsidence of the disorder, were in inverse proportion to the age of the subject—a child eight or ten months old bearing much larger proportionate doses, than one from eight to ten years.

When the pupils have become dilated, the dose was diminished for a few days.—*Lancet.*

---

Digitalis in the Treatment of Epilepsy.—A nursing child, not quite two years old, was brought to Prof. Clark's clinic, to be treated for "fits," from which it had suffered for the last twelve months, occurring every three or four weeks, limited to one a day, though on one day it had seven.

The character of the disease was evidently epileptic, and Professor Clark determined to give the digitalis a trial. The child was accordingly put upon one drop of the tincture three times a day, with directions to increase the dose gradually, as circumstances might indicate. No attack occurred, however, since commencing with the tincture, one drop of which had been taken regularly, three times a day, until four months had elapsed, when the child was last seen at the clinic.

Prof. Van der Kock has had some success in the treatment of epilepsy, by applying cupping glasses, with scarification, or leeches, to the back of the neck, followed by seton, or issue, with a view to moderate the exalted sensibility of the medulla oblongata, and prescribing internally the infusion of digitalis, with small doses of tartar emetic, if the patient can bear them without nausea, to moderate still further the excited vascular action; but he says he never succeeded in curing a case with digitalis alone, though he believes it contributes much towards promoting the cure.—*Cincinnati Lancet & Observer.*

---

Oxygen Gas.—At a recent meeting of the Academy of Sciences at Munich, Baron Liebig recounted various experiments which proved clearly that oxygen is not only evolved from the atmosphere by plants, but also in tolerably large quantities by decomposition of water in the bodies of flesh-eating animals. He thinks that a knowledge of this fact will throw quite a new light on the processes of nutrition and digestion.—*London Lancet.*

---

Professor Trousseau on Aphasia.—This popular professor, in one of his late clinical lectures at the Hotel Dieu of Paris, dwelt on a peculiar complaint, the symptoms of which are the inability in the patient pronouncing certain words, and of expressing his thoughts, retaining at the same time the full use of his intellectual faculties. M. Broca, who first directed attention to this malady, called it *aphœmia,* which term M. Trousseau, aided by the celebrated philologist, Littre, considers wrong, as it really means "bad repute." *Aphasia* is better.—*Ib.*

---

### SYMPTOMS OF POISONING FOLLOWING A DOSE OF DISULPHATE OF QUININE.

*To the Editor of* The London Lancet.

Sir:—I beg to forward the accompanying case, as I think it may prove interesting to many members of the profession:—

I was called in great haste one evening to a case of supposed poisoning. On arriving at the house, I found the man much better, but thought it safer to administer an emetic. On making inquiries, I found that he had prescribed for himself a mixture of sarsaparilla and quinine, which he had purchased from a druggist. The symptoms of poisoning came on after the first dose of this mixture: within a few minutes there was severe pain and burning in the stomach; the face swelled; the mouth felt drawn forwards; then the legs and body swelled, and became very red, with intolerable itching, followed by a rash of urticaria. I thought it possible that some poison had become accidentally mixed with his mixture, and so the case rested for a time. However, shortly afterwards the man had an attack of pneumonia, and during his convalescence quinine was prescribed. Upon taking the first dose (two-thirds of a grain, Howard's), all the symptoms above described came on, clearly proving that the quinine was the original cause of the mischief.

I think this case interesting from the singularity and violence of the symptoms produced by so small a dose, and also as showing how a druggist may be unjustly blamed from an idiosyncrasy of a patient.

I am, Sir, your obedient servant,

E. H. ROE.

---

### UTERINE ACTION DURING SLEEP.

---

*To the Editor of* THE LONDON LANCET.

SIR:—I have read with great interest Dr. Palfrey's case in reference to the above subject, and, in confirmation of his opinion, beg to forward you the following statement:—

I was called on the morning of the 20th of October last to see a lady in her second confinement. Her residence being within a few doors of my own, no time was lost in visiting her. I found the child's head resting upon the perineum, and in a few minutes she was safely delivered of a fine healthy son. On going to bed the previous night she felt quite well, and had no intimation of the event about to take place, except a slight discharge, of which she took no great notice.

In this case, there is no doubt that the whole stage of dilatation, and partly that of expulsion, had taken place during sleep, as from her awaking until the birth of the child no longer period than half an hour could have elapsed. Her first confinement some twelve months previously had been very severe, occupying some forty-eight hours, with delivery by forceps. In this instance there were only three or four labor pains, and no after-pains whatever.

I am, Sir, your obedient servant,

JOHN HARVEY, M.D.

